# Characterisation of rollator use using inertial sensors

**DOI:** 10.1049/htl.2016.0061

**Published:** 2016-11-02

**Authors:** Tsu-Jui Cheng, Laurence Kenney, James David Amor, Sibylle Brunhilde Thies, Eleonora Costamagna, Christopher James, Catherine Holloway

**Affiliations:** 1 Centre for Health Sciences Research, University of Salford, Salford M6 6PU, UK; 2 Warwick Engineering in Biomedicine, School of Engineering, University of Warwick, Coventry CV4 7AL, UK; 3 Department of Computer Science, University College London, Gower Street, London WC1E 6BT, UK

**Keywords:** rollers (machinery), inertial navigation, handicapped aids, geriatrics, medical disorders, biomedical measurement, rollator use, inertial sensors, walking aids, mobility impairment, outdoor mobility, curbs, slopes, urban environment, rollator movement, inertial measurement unit, push events, motion capture system, multiple sclerosis, IMU-derived metrics, outdoor environment

## Abstract

The use of walking aids is prevalent among older people and people with mobility impairment. Rollators are designed to support outdoor mobility and require the user to negotiate curbs and slopes in the urban environment. Despite the prevalence of rollators, analysis of their use outside of controlled environments has received relatively little attention. This Letter reports on an initial study to characterise rollator movement. An inertial measurement unit (IMU) was used to measure the motion of the rollator and analytical approaches were developed to extract features characterising the rollator movement, properties of the surface and push events. The analytics were tested in two situations: first, a healthy participant used a rollator in a laboratory using a motion capture system to obtain ground truth. Second, the IMU was used to measure the movement of a rollator being used by a user with multiple sclerosis on a flat surface, cross-slope, up and down slopes and up and down a step. The results showed that surface inclination and distance travelled measured by the IMU have close approximation to the results from ground truth; therefore, demonstrating the potential for IMU-derived metrics to characterise rollator movement and user's pushing style in the outdoor environment.

## 1 Introduction

In the United States ∼4.2 million older adults use at least one walking aid, with a view to reducing fall risk and/or enhancing mobility [1]. A European study that included the UK found that walking aids were reported to be used by 29–49% of older people [2]. However, as will be discussed in more detail below, we have surprisingly little objective data on the extent to which such devices are actually used, how they enhance mobility or reduce fall risk. Indeed, a rather surprising finding from a number of studies is that their reported use has been associated with falls. Research found that hospitalised patients who fell were more likely to be users of walking aids [3], and a meta-analysis associated walking aid use with a two–three-fold risk of falling [4]. Whilst correlation cannot be assumed to indicate causation, this is certainly of serious concern and justifies further research.

Rollators are the most and second most common walking aids in Sweden [5] and Canada [6], respectively, due to the greater provision of stability support than walking sticks. Rollators are often fitted with seats and/or baskets to allow users to travel longer distances and run errands outdoors. Rollators typically have manual brakes installed on the rear wheels to prevent the rollator running away from the user while the user is moving and also to allow the user to adjust the movement of rollator in relation to their gait pattern.

A small number of studies have reported on user views on rollators. Brandt *et al.* [7] carried out a longitudinal study using the Quebec User Evaluation of Satisfaction with Assistive Technology (QUEST version 1) to understand the satisfaction with rollators among community-dwelling users (mean age of 76) in Denmark. The overall satisfaction with rollators was above 90%, particularly with the effectiveness, durability and safety of rollators. More than two-thirds of the users reported using their rollators at least once a day. However, rollators were reported to be too heavy to handle when getting over curbs and steps. A study by Lindemann *et al.* [8] found that rollator users reported walking downhill, uphill, over uneven surfaces outdoors and obstacle crossing to be major concerns with regard to safety. Rollator users in Denmark [7] and Japan [9] were found to be less satisfied with the professional and follow-up services including the provision of training by the physiotherapists, repairs and visits. This left them without enough knowledge of basic instructions, adjustments to and repairs of their rollator. In addition, there was a lack of channels to feedback or report problems with their rollator.

From a biomechanics perspective, despite their prevalence amongst the older population, the literature on characterisation of rollator–user interaction is very limited. Kegelmeyer *et al.* [10] studied 27 individuals with Parkinson's disease, finding that rollator use led to less variability in gait measures of velocity, stride length, per cent swing and double support time compared with walking sticks, walking frames, two-wheeled walkers and U-Step walkers. Lindemann *et al.* [8] studied the gait of 22 rollator users (median age of 82) in a geriatric rehabilitation clinic in Germany. The results showed that with rollators, users walked faster with smaller step width and higher walk ratio (i.e. step length divided by step frequency) than without rollators in both forward and backward walking, indicating an improved walking performance. However, complex walking tasks such as opening a door were found to lead to the impossibility to open and pass through a door with a rollator, because of the rigid rear wheels. Chee *et al.* [11] investigated the step width, the variability of step width and velocity of two community-dwelling rollator users with multiple sclerosis (MS) by comparing their performance in the laboratory and outdoor walking environment including an urban pavement, a ramp and pedestrian crossing, using an instrumented rollator. The results suggest that the outdoor walking environment may affect foot placement patterns, and hence potentially, trip risk. The step-width variability of up-ramp walking had greater step-width variability than laboratory walking and down-ramp walking, indicating an unstable mediolateral movement which could lead to falls. Moreover, the walking velocity significantly increased at the pedestrian crossing as compared with walking in the laboratory.

In one of the most recent papers, Tung *et al.* [12] studied three stroke or traumatic brain injury users of rollators in the laboratory and on a walking course inside a rehabilitation hospital containing hallways, turns, ramps, doors and lifts. A single-axis load cell was mounted into each leg and a three-axis accelerometer was mounted under the seat of the rollator to capture the performance of rollator use. High fall risk behaviours such as collisions with door frames and between the foot and the rollator, as well as stumbling and lifting the rollator, were observed in the walking course.

Despite the recent advances in low-cost computing and sensing, there is no data on the patterns of use of rollator devices outside of controlled environments, whereas in other areas of mobility aids research such as wheelchairs the usage and activity levels can be measured by accelerometers and inertial measurement units (IMUs) [13–15]. This is very surprising, particularly given the high prevalence of rollators amongst older people and recent studies that indicate the potential for increased trip or fall risk outside of the laboratory [11, 12]. Indeed, even basic information on the extent to which prescription of such devices leads to increased mobility is absent.

In the light of this, this Letter reports on a feasibility study to characterise rollator use in the laboratory using real-world surfaces. Two experiments are presented, first an experiment with a healthy user, and second an experiment with a user who has MS. The first experiment demonstrates how a single IMU mounted on the rollator frame together with sensors on the user's feet can be used to characterise basic features of rollator use. These features are number of push events, distance travelled, average distance and duration of each push, and the push events in relation to the foot movements. The second experiment applies this technique to one rollator user with MS in a simulated urban environment (SUE) and demonstrates the potential to obtain information on the environment including surface slope and curb crossing events from a rollator-mounted sensor, in addition to the basic gait features.

## 2 Methods

The aim of the feasibility study was to establish the capability of the IMU to capture the interaction between the rollator, the user and the walking environment. To evaluate the capability of the IMU, the experiment was two-fold, containing (i) testing of protocols and software algorithm using a gold standard motion capture system and (ii) testing of the protocols and algorithm in an SUE.

### 2.1 Participants

A healthy participant was recruited for understanding baseline performance. Subsequently, a participant with 3 years of MS participated in tasks in the SUE. Ethical approval was obtained from the University College London Research Ethics Committee (4721/002).

### 2.2 Gold standard testing in the laboratory

**Fig. 1:**
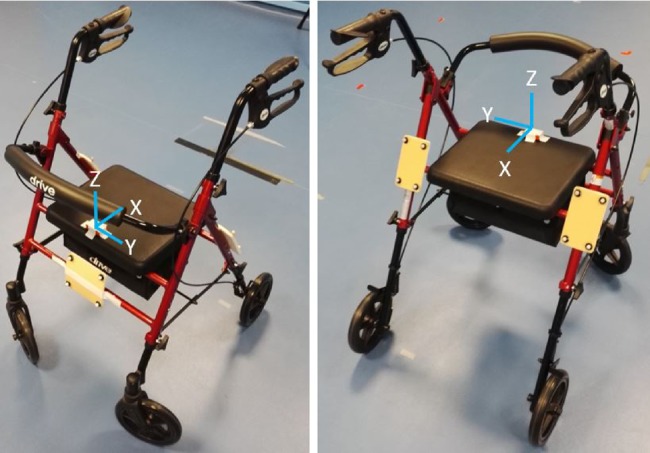
Placement of the IMU, taped in white and on top of the seat and cluster markers, on the left, right and front side of the rollator

The gold standard test comprised a 6 m straight-line walking assessment with a rollator. The healthy participant had IMUs of Xsens MTw2 Awinda (Xsens Technologies Besloten Vennootschap, the Netherlands) attached to the pelvis and both feet, operating at a sampling frequency of 100 Hz. To obtain ground truth, the three-dimensional coordinate data of the pelvis and both feet were captured using an eight-camera VICON Motion Capture System at a sampling frequency of 100 Hz. On the rollator, there were an IMU horizontally attached to the frame and a cluster of markers to each of the left, right and front side of the frame as shown in Fig. 1. The orientation of the IMU on the rollator is the *Y*-axis for anterior–posterior movements, the *X*-axis for mediolateral movements and *Z*-axis for vertical movements. The IMU is oriented such that a negative value in the *Y*-axis corresponds to forward movement. The rollator was banged onto the force plates by the participant before the start of each trial to get a peak force in both VICON and Xsens to synchronise the two datasets.

### 2.3 Testing in the SUE

**Fig. 2:**
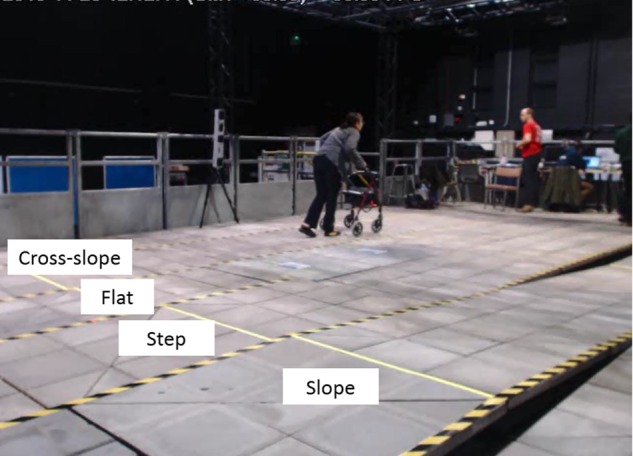
Experiment set-up for the SUE and the property of surfaces

The tests in the simulated environment used the same IMU placement as the gold standard testing, but did not use the motion capture system. The tests consisted of a participant moving along four straight lanes including an 8.4 m flat path, an 8.4 m 4% cross-slope (2.29° elevation across the distance of travel), a 4.8 m 6% slope (3.44° elevation in the distance of travel) and a step of 80 mm, which were set up at the Pedestrian Accessibility Movement Environment Laboratory at University College London as shown in Fig. 2. The participant with MS was asked to move along each lane at a self-selected speed and in a way they normally moved in their everyday environment. In each lane, the participant performed one to three trials, depending on their physical capability, with a pre-experiment in which several trials were conducted to familiarise the user with the laboratory settings.

## 3 Data analysis

The results of the gold standard testing from the motion capture system served as the ground truth to examine the analysis of the IMU data for distance travelled, whilst the known characteristics of the surface of the SUE served as ground truth for surface detection. Gait phase data was obtained from the foot-worn IMU, which was measured alongside the push events of the rollator. The analysis was utilised to measure the characteristics of rollator usage in the laboratory and SUE.

### 3.1 Raw data and filtering

The raw data in the ***X***, ***Y*** and ***Z*** axes are vectors with length *n* of the form

$${\bi X} = \left({x_1\comma \; \, x_2\comma \; \, x_3\comma \; \, \ldots \comma \; \, x_n} \right).$$

$${\bi Y} = \left({y_1\comma \; \, y_2\comma \; \, y_3\comma \; \, \ldots \comma \; \, y_n} \right).$$

$${\bi Z} = \left({z_1\comma \; \, z_2\comma \; \, z_3\comma \; \, \ldots \comma \; \, z_n} \right).$$

Two different filtering operations are applied to the data prior to subsequent processing, a low-pass filter and a band-pass filter, to give two differently filtered versions of the raw data. A fourth-order Butterworth low-pass filter at 0.2 Hz is used to extract the baseline from the data, as ${\bi X}^b$, ${\bi Y}^b$ and ${\bi Z}^b$, with components $x_i^b $, $y_i^b $ and $z_i^b $. A second-order Butterworth band-pass filter between 0.2 and 3 Hz is used to extract the motion-related component of the signal as ${\bi X}^m$, ${\bi Y}^m$ and ${\bi Z}^m$, with components $x_i^m $, $y_i^m $ and $z_i^m $.

### 3.2 Surface detection

The acceleration of the *Y*-axis is used to calculate the longitudinal tilt of the rollator on the flat surface, slope and step; the acceleration of the *X*-axis is used to calculate the cross-sectional tilt of the rollator on the cross-slope.

The low-pass data are used for surface detection. From these data, the orientation of the rollator with respect to the gravitational pull of the earth is estimated, which provides the angle of the horizontal plane of the rollator to the earth. From this angle the direction of the surface slope, if any, can be determined. Orientation is calculated as

(1) $$\theta _i^Y = \cos ^{ - 1}\left({\displaystyle{{y_i^b } \over {\sqrt {x_i^{b^2} + y_i^{b^2} + z_i^{b^2} } }}} \right)\comma \; \eqno\lpar 1\rpar $$

(2) $$\theta _i^X = \cos ^{ - 1}\left({\displaystyle{{x_i^b } \over {\sqrt {x_i^{b^2} + y_i^{b^2} + z_i^{b^2} } }}} \right)\comma \; \eqno\lpar 2\rpar $$

### 3.3 Distance travelled

Distance travelled is obtained principally from a double integration of the accelerometer signal in the direction of travel. For this work, only the *Y*-axis (corresponding to the anterior–posterior orientation of the rollator) has been used. This axis is oriented approximately parallel to the ground in the direction of movement and thus captures the majority of the motion of interest.

The band-pass filtered data were used to calculate distance travelled. After filtering, the signal was cumulatively, numerically integrated to obtain velocity over time, ${\bi Y}^v$. This is achieved using the trapezoidal rule for integration, given in this case as

(3) $$f\left({{\bi Y}\comma \; \, a\comma \; \, b} \right)= \int_a^b {\bi Y} = \; \displaystyle{{b - a} \over {2\left({b - a} \right)}}\mathop \sum \limits_{i = a}^{b - a} y_i + y_{i + 1}\eqno\lpar 3\rpar $$

where *a* and *b* are the indices of ***Y*** between which an integral is required. Equation (3) is then used cumulatively to provide the cumulative numeric integration as

(4) $$\eqalign{g\left({{\bi Y}\comma \; \, \alpha \comma \; \, \beta } \right)&= \left({\,f\left({{\bi Y}\comma \; \, \alpha \comma \; \, \alpha } \right)\comma \quad f\left({{\bi Y}\comma \; \, \alpha \comma \; \, \alpha + 1} \right)\comma \quad}\right. \cr &\quad f\left({{\bi Y}\comma \; \, \alpha \comma \; \, \alpha + 2} \right)\comma\; \, \ldots \comma \; \, f\left({{\bi Y}\comma \; \, \alpha \comma \; \, \beta } \right).}\eqno\lpar 4\rpar $$

Owing to the high-pass filtering removing the DC component, the velocity oscillated around zero, which transposed the velocity downwards, which when integrated to get distance results in error building up cumulatively. To counteract this, an adjustment was made to the velocity signal based on the assumption that a person pushing a rollator will not maintain a constant velocity unless the rollator is stationary. Therefore, if the stationary periods are identified, the velocity signal can be zeroed around these points to get back to true velocity.

To achieve this, a baseline signal is created by interpolating between velocity points where the gradient is below 0.5 × 10^−3^. The set of zero-points and their associated timestamps are interpolated to get a baseline signal with the same timestamps as the velocity signal using MATLAB's pchip interpolation, which is based on work by Fritsch and Carlson [16] and Kahaner *et al.* [17]. Pchip interpolation was chosen as it is only based on points close to the interpolation target and is robust to local changes in signal.

Once a baseline signal is created, it is added to the velocity signal to correct the offset. The adjusted velocity signal is then cumulatively integrated a second time, using (4), to get distance travelled. Other parameters of interest such as push identification can be obtained from a simple analysis of the adjusted velocity signal or the cumulative distance travelled.

## 4 Results

### 4.1 Gold standard testing in the laboratory

**Fig. 3:**
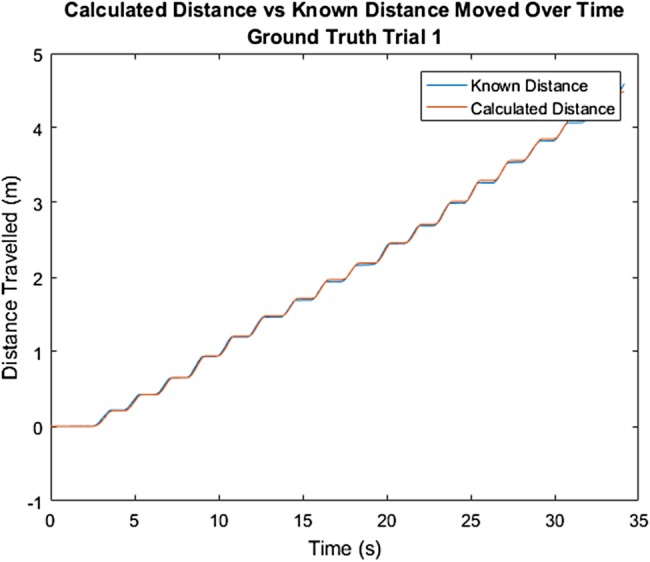
Comparison between distance calculated by IMU and known distance from motion capture system in the gold standard testing with the healthy participant

**Fig. 4:**
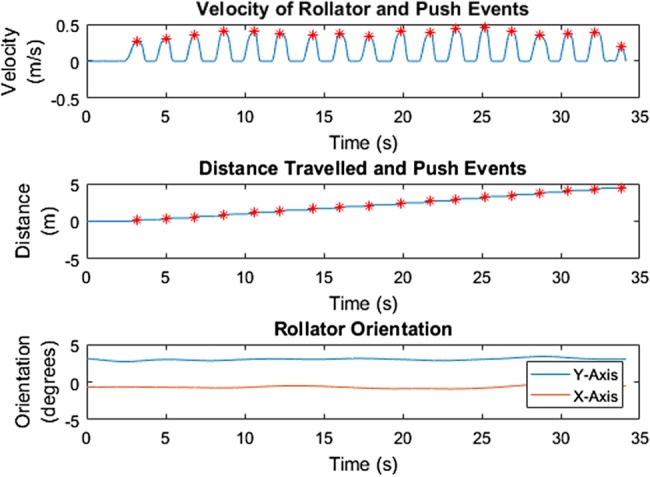
IMU data shows velocity (top) and distance travelled (middle) in relation to push events (red stars); and the orientation of the rollator over time (bottom) in the gold standard testing with the healthy participant

Results from the ground truth test with the healthy participant showed that calculated distance travelled is a very close approximation to ground truth for both tests. Fig. 3 shows this for one of the two tests. Furthermore, a distinct push pattern, as shown in Fig. 3, can be identified. Fig. 4 shows the derived velocity signal, cumulative distance and orientation of the rollator over time. Pushes, identified as moments of peak positive velocity, are identified with red stars. The orientation of the rollator shows a constant orientation over the walk, indicating no change in orientation occurred.

**Fig. 5:**
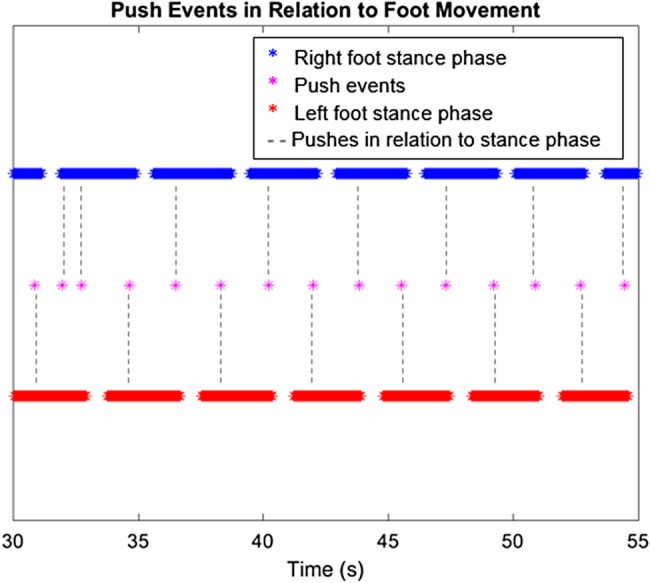
Push events from IMU data in relation to foot movement in the 25 s segment in the gold standard testing with the healthy participant

The basic features of rollator use of the healthy participant including the number of push events, average distance and distance of each push, and mean velocity of rollator movement are shown in Table 1. Fig. 5 shows a distinctive pattern of a push event happening around the start of a stance phase of either of the feet, demonstrating the healthy participant's pushing style.

**Table 1:** Basic features of rollator use including number of push events, average distance and distance of each push, and mean velocity of rollator movement

Surface types	Number of push events	Average distance per push, m	Average duration per push, s	Mean velocity, m/s
gold standard with the healthy participant	–	–	–	–
4.6 m flat surface	18	0.2623	1.8062	0.1459
SUE with the MS participant	–	–	–	–
8.4 m flat surface	36	0.2344	1.7118	0.1367
4% 8.4 m cross-slope (right)	39	0.2311	1.3989	0.1516
4% 8.4 m cross-slope (left)	19	0.3105	1.6447	0.1904
6% 6 m up-slope	38	0.1904	1.4522	0.1283
6% 6 m down-slope	19	0.3248	1.6765	0.1952
80 mm step-up on 8.4 m path	45	0.1973	1.5656	0.1284
80 mm step-down on 8.4 m path	43	0.2154	1.5546	0.1423

### 4.2 Testing in the SUE

The basic features of rollator use of the MS participant measured by the analysis of IMU data developed in the gold standard testing and applied to the SUE data are presented in Table 1.

**Fig. 6:**
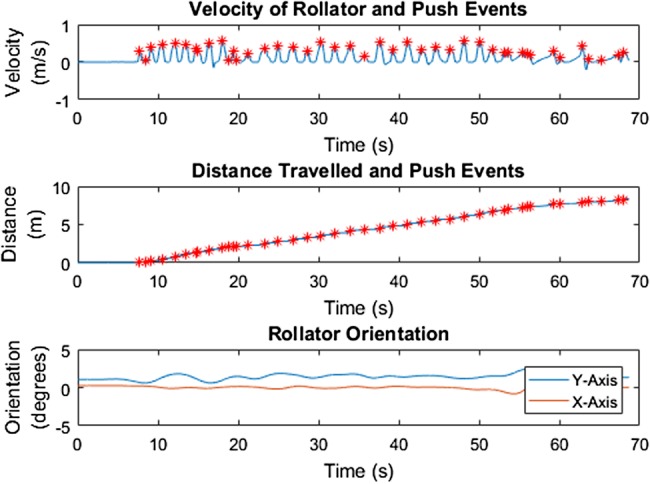
IMU data shows velocity (top) and distance travelled (middle) relating to push events; the orientation of the rollator (bottom) along the flat surface with the MS participant in the SUE

**Fig. 7:**
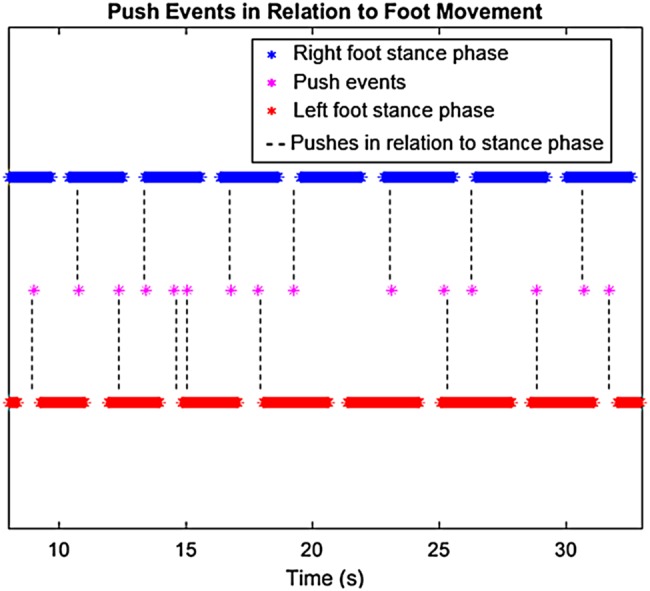
Push events from IMU data relating to foot movement in the 25 s segment along the flat surface with the MS participant in the SUE

**Fig. 8:**
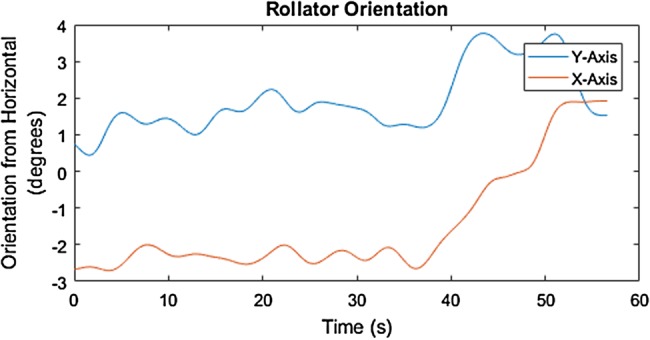
Orientation from IMU data, between 0 and 37 s, of the rollator along the 4% (2.29°) cross-slope with the elevation on the right with respect to the MS participant in the SUE

**Fig. 9:**
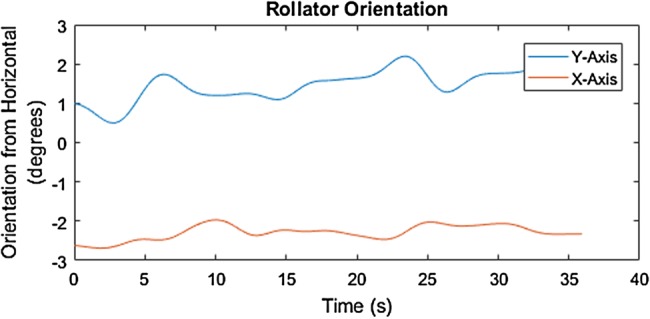
Orientation from IMU data of the rollator along the 4% (2.29°) cross-slope with the elevation on the left with respect to the MS participant in the SUE

**Fig. 10:**
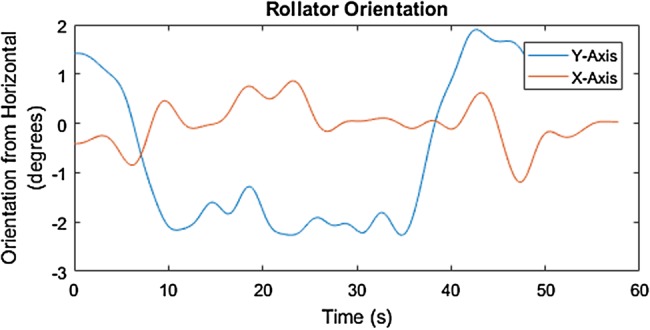
Orientation of the rollator along the 6% (3.44°) up-slope with the MS user in the SUE

**Fig. 11:**
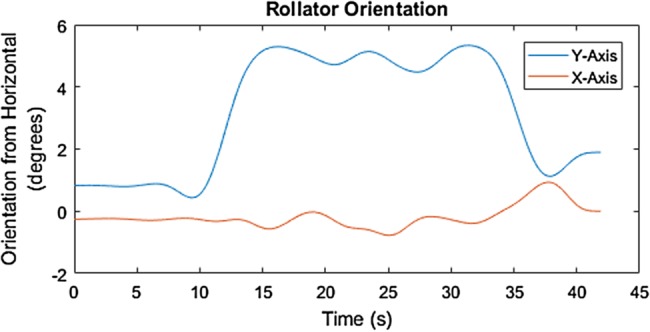
Orientation of the rollator along the 6% (3.44°) down-slope with the MS user in the SUE

Similar to the results in the gold standard testing, the characteristics of the rollator movement of the MS participant along the flat surface are comparatively steady, as shown in Fig. 6, as opposed to other surfaces as shown in Figs. 8–11. Results from the simulated surface testing on the flat surface, as shown in Fig. 6, are encouraging with total distance travelled from IMU data being approximately equal to the known distance measured by the motion capture system. The push pattern is harder to identify in this data, but is likely to be the result of the MS participant's particular gait pattern. The pushing style also demonstrates a similar pattern to the gold standard testing in which a push event happened around the start of a stance phase, as shown in Fig. 7.

The mediolateral inclination of the rollator movement along the 6% cross-slope is identified by the degrees elevation across the distance of travel, around −2° to −3° on the *X*-axis, as shown in Figs. 8 and 9. The start and end of the 6% slope is identified by the change in degree elevation from around −2 to +5 on the *Y*-axis (Figs. 10 and 11).

**Fig. 12:**
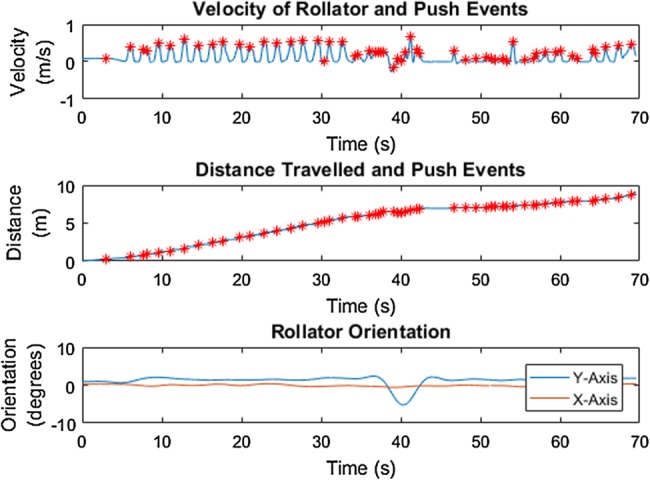
IMU data shows velocity (top) and distance travelled (middle) in relation to push events; the orientation of the rollator over time (bottom) during the step-up with the MS participant in the SUE

**Fig. 13:**
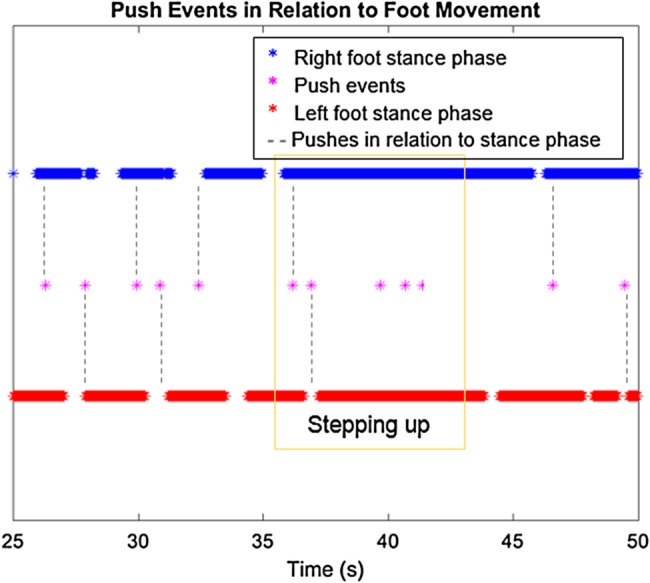
Push events from IMU data for foot movement in the 25 s segment during the step-up with the MS participant in the SUE

During the step-up and step-down, the regular movement of the rollator is shown to have been interfered with the step. Figs. 12 and 13 show an increase of push events when the MS participant was encountering the step-up. The orientation data in Fig. 12 suggests that the rollator might be initially pulled close to the MS participant and then lifted up to the raised step, hence a dip in the orientation in the *Y*-axis.

**Fig. 14:**
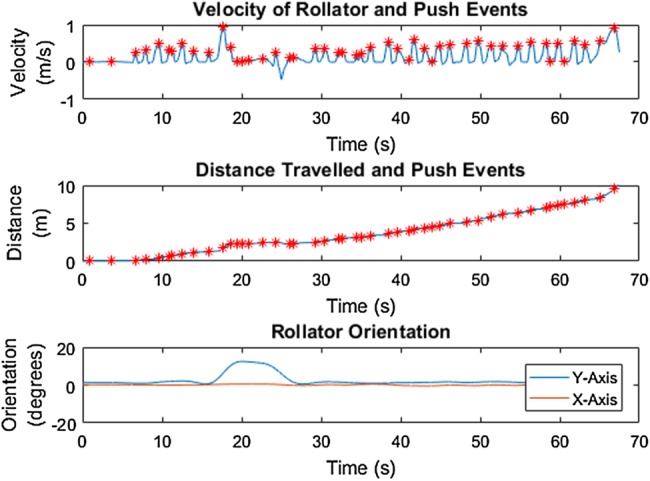
IMU data shows velocity (top) and distance travelled (middle) in relation to push events; the orientation of the rollator over time (bottom) during the step-down with the MS participant in the SUE

**Fig. 15:**
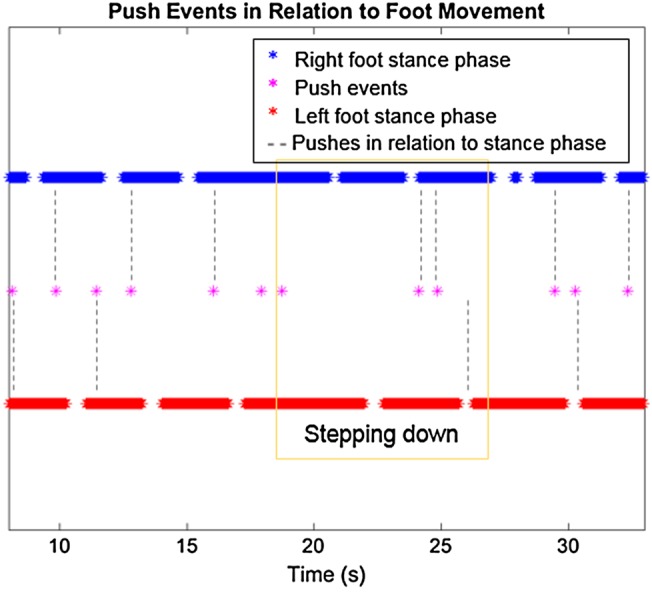
Push events from IMU data relating to foot movement in the 25 s segment during the step-down with the MS participant in the SUE

Figs. 14 and 15 show an increased interval between pushes when the MS participant was encountering the step-down. The orientation data in Fig. 14 suggest that the rollator might be pushed away from the MS participant and then land on the lowered step, hence the peak in the orientation of the *Y*-axis.

## 5 Discussion

The results of the tests in the laboratory and SUE show that it is feasible to use an IMU to characterise the rollator movement and measure the interaction between the rollator, the user and the urban environment. The results also show that by using an IMU alone, the travel pattern can be reconstructed offline, which can provide researchers and physiotherapists with insight into a user's performance while walking and using a rollator.

Past studies have demonstrated the difference in the movement behaviour between laboratory assessments and real environment and call for a better understanding of the interaction [11, 12]. This Letter clearly demonstrates, the healthy participant's pushes, distance travelled, average distance and duration of each push, and the push events in relation to the foot movement in the laboratory through the motion capture system and IMU. When the IMU and protocol were then brought to the SUE, the MS participant can be seen to tend to consistently initiate the push of the rollator around the heel strike of each foot. The MS participant demonstrates a smooth and less interfered gait with the help of the rollator, which has also been shown in past studies [8, 10]. However, the MS participant's movement was interfered while walking up the step due in part to the physical constraint of lifting the rollator up or down the step. This is also a type of collision in the urban environment that past studies [11, 12] indicated and this Letter has demonstrated the capability of IMUs to record and measure the foot and rollator movements during these collisions.

The property of the surface and distance travelled can be detected by the IMU by the degree of the inclination of the rollator and integration of the acceleration of the rollator movement. Along with the push events in relation to the foot movement and average distance and duration of each push, the user's balance mechanism and coping strategy used to deal with the uneven surface in the urban environment can be further understood.

Investigating the characterisation of rollator use has helped shed some light on the understanding of the quality, difficulty and risk of the use of rollators in the urban environment. Furthermore, studies need to investigate how the understanding of this interaction between the rollator, the user and the urban environment can help physiotherapists provide training, rehabilitation and assessments for rollator users of different physical, cognitive and sensory capabilities.

We do, however, acknowledge several limitations of this Letter. As a pilot study exploring the interaction between the rollator, the user and the environment, only one participant was measured in each of the laboratory and SUE. This Letter does not intend to demonstrate the generalisability of findings, but explore the potential and validation of using low cost, portable IMUs to characterise rollator use outside the laboratory setting. This Letter provides initial evidence to conduct future research with larger sample sizes, more types of surfaces and longer walking distances. Furthermore, work will focus on creating a generalised set of algorithms to extract rollator characterisation data from IMUs and the applications of this approach to different user groups.

## 6 Conclusions

The work presented in this Letter provides a first examination of the interaction between the rollator, the user and the environment using potable IMUs to characterise the rollator movements. A healthy participant performed walking tests using a rollator on a flat surface in the laboratory to examine the IMU measures with the gold standard ground truth from a motion capture system. Subsequently, a participant with MS performed walking tests using a rollator on a flat surface, cross-slope, up and down-slope and up and down a step in an SUE with an IMU alone attached. The use of IMUs to measure the pushing style, property of surface and travel distance has been examined by the motion capture system and can be utilised to detect these movement characteristics of a rollator user with MS on different surfaces. The results of this Letter show the potential to provide insight into the quality of the use of rollators, fall risks associated to rollators and quality of the provision of rehabilitation for rollator users.
